# Analysis between preoperative cervical radiographic parameters represented by the K-line tilt and the short-term prognosis of laminoplasty for posterior longitudinal ligament ossification: A retrospective study

**DOI:** 10.3389/fsurg.2022.950707

**Published:** 2022-09-22

**Authors:** Baixing Wei, Wanting Liu, Han Wu

**Affiliations:** ^1^ Department of Orthopedics, China-Japan Union Hospital of Jilin University, Changchun, China; ^2^ Department of Clinical Medicine, First Affiliated Hospital of Harbin Medical University, Harbin, China

**Keywords:** ossification of the posterior longitudinal ligament, laminoplasty, cervical radiographic parameters, K-line, quality of life

## Abstract

**Objectives:**

To investigate the relationship between preoperative radiographic parameters and the short-term prognosis of patients with cervical ossification of the posterior longitudinal ligament (OPLL) who underwent laminoplasty (LAMP).

**Methods:**

A retrospective analysis of Cervical OPLL 50 patients with K-line (+) OPLL with no cervical kyphosis who received LAMP was performed. Based on preoperative neutral position x-ray, the K-line tilt, C2–C7 SVA (sagittal vertical axis), CL (cervical lordosis), T1 slope, and T1 slope-CL were recorded. The JOA (Japanese orthopaedic association scores) score and the cervical kyphosis change were recorded 1 year after surgery. Patients were divided into good and poor prognosis groups according to the median (12.5) of the postoperative JOA score.

**Results:**

There were differences between the two groups in K-line tilt, C2–C7 SVA, and T1 slope (all *p*s < 0.05). There was a strong linear correlation between the three, K-Line tilt, JOA score, and C2–C7 SVA. The degree of influence of K-line tilt, C2–C7 SVA, T1 slope on postoperative JOA score was analyzed using multiple linear regression, and the absolute value of the standardized coefficient Beta were 0.550, 0.319, 0.185, respectively. There was no cervical kyphosis change 1 year after surgery.

**Conclusion:**

As preoperative cervical parameters, the influence of K-line tilt, C2–C7 SVA, and T1 slope on postoperative JOA score decreases in order. There was a linear relationship between preoperative K-line tilt and postoperative JOA score, implying that patients with cervical OPLL with high K-line tilt were not eligible for LAMP. K-line tilt was not predictive of cervical kyphosis change after LAMP in patients with OPLL at short-term follow-up.

## 1. Introduction

Ossification of the posterior longitudinal ligament (OPLL) is a common disease leading to spinal or radicular cervical spondylosis. It has a prevalence of 1.9%–4.3% in the East Asian population (1). This disease frequently necessitates surgical treatment. The most common surgical approaches are (2): anterior decompression with fusion (ADF), posterior decompression with fusion (PDF), combined anterior and posterior decompression with fusion (APDF) (3), and double/single-door laminoplasty (LAMP) (4). Although ADF decompression is more direct and complete in patients with severe spinal canal encroachment, the posterior decompression technique, especially LAMP, is more commonly used in clinical practice due to its simplicity, safety, and low complication rate (5, 6).

Fujiyosh (7) defined K-line as a straight line drawn from the midpoints of the C2 and C7 spinal canals on a neutral lateral x-ray. When the ossified part does not exceed the K-line, it is referred to as K-line (+), and when it does, it is referred to as K-line (−). In the study, Fujiyosh (7) discovered that K-line (−) patients who underwent LAMP had a poor prognosis due to insufficient decompression. Furthermore, preoperative K-line (+) patients recovered neurologically better than preoperative K-line (−) patients in cervical OPLL patients who underwent LAMP (8).

K-line, C2–C7 cervical sagittal vertical axis (C2–C7 SVA), T1 slope, cervical lordosis (CL), and T1 slope-cervical lordosis (T1 slope-CL) are all cervical radiographic parameters measured preoperatively on neutral lateral x-ray. These parameters can predict the prognosis of cervical spine surgery (9, 10).

Kim (11) introduced the K-line tilt in 2018, and it is a derivative form of K-line, defined as an angle between the K-line and the plumb line of the horizon. Combined with the findings of subsequent studies (12–14), K-line tilt can be used as a new preoperative cervical radiographic parameter to predict cervical spine surgery prognosis.

Since there have been few studies on K-line tilt, the primary goal of this study was to investigate the relationship between the preoperative K-line tilt and the short-term prognosis of patients with cervical OPLL who underwent LAMP, with prognosis measured by the JOA score, as well as the correlation with other preoperative cervical radiographic parameters.

## 2. Materials and methods

### 2.1. Ethics and patient consent

This study was approved by the Ethics Committee of the China-Japan Union Hospital of Jilin University. All the patients provided written informed consent in this study.

### 2.2. Materials

The study design was a retrospective collection of data of patients who underwent LAMP for cervical OPLL at the Department of Spine Surgery, China-Japan Union Hospital, between January 2017 and December 2020. Their preoperative and postoperative clinical and imaging data were also collected. All patients were diagnosed after a thorough medical history interview, physical examination, and imaging.

### 2.3. Criteria for acceptance and exclusion

#### 2.3.1. Criteria for acceptance

(1) Complete preoperative and follow-up radiological data, all preoperative radiological data within 2 weeks, postoperative follow-up data 1 year after surgery; (2) x-ray diagnosis of cervical OPLL with K-line (+) and no cervical kyphosis; (3) preoperative CT determined that OPLL involved two or more vertebrae; (4) preoperative CT/MRI revealed cervical spinal stenosis and significant spinal cord compression; (5) physical examination revealed clear signs and symptoms of spinal cervical spondylosis, necessitating LAMP treatment and anticipated neurological recovery from surgery.

#### 2.3.2. Criteria for exclusion

(1) History of cervical spine surgery; (2) patients with severe underlying diseases; (3) combined cervical spine trauma and spinal cord injury; (4) combined psychiatric disorders preventing treatment and follow-up; (5) patients with severe combined osteoporosis; (6) exclusion of patients with difficulty measuring x-ray parameters (e.g., Obstruction of T1 vertebrae due to sternal or rib obstruction or obesity); (7) Tumors of the cervical spine.

### 2.4. Surgical methodology

**Figure 1:**
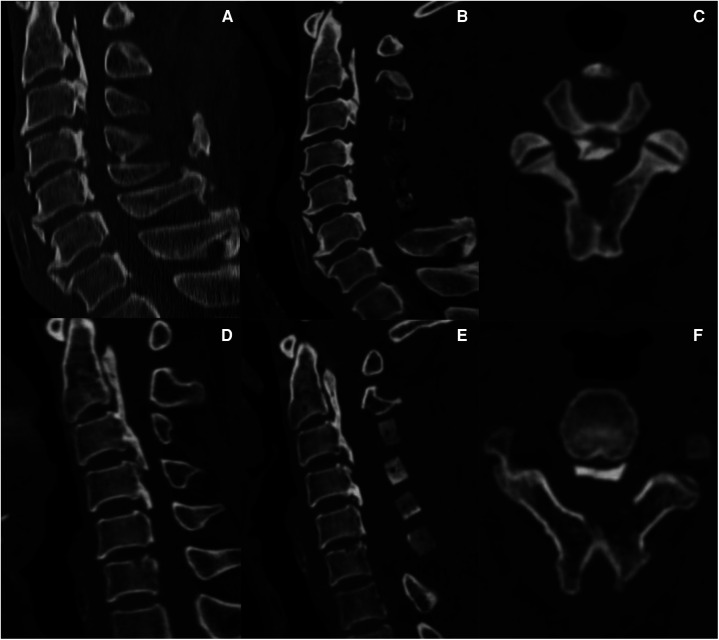
Dome-like Expansive LAMP for C2 decompression.

The Surgical approach was performed using the LAMP described by Tomita (15). Separate the paravertebral muscles from the spinous processes to expose the laminae. If the operating segment was C3–C6, the procedure was as follows: when laminae were separated, and the ligamentum flavum of C2–C3 and C6–C7 was excised to expose the midline epidural space for decompression. A wire saw was inserted from the C6–C7 midline epidural space and threaded through C2–C3 midline epidural space. The spinous process was split centrally by repeated pulling with the wire saw. The outer layer of cortical bone and some cancellous bone on the medial side of the articular eminence on both sides of C3–C6 were removed using a high-speed air-burr drill. They were made into V-shaped bone grooves on both sides of the laminae which served as the portal axes of the double doors. The bone cortex on either side of the groove was incompletely fractured. The residual ligamentum flavum and epidural adhesions were also removed. The spinal canal was sufficiently enlarged. Using the high-speed air-burr drill, holes were created in the root of the split spinous process on both sides. Four trapezoidal homogeneous bone blocks were fixed to the C3–C6 bilateral spinous processes with double 10-gauge wire, and the knots were checked and firmly fixed. When faced with C2 decompression, we performed Dome-like Expansive LAMP (16, 17) (Figure 1). Briefly, a drill was applied to make a dome-like groove on the caudal surface of the C2, removed part of the anterior portion of the laminae, and then carefully excised the excess hypertrophic ligamentum flavum and part laminae. When faced with C7 decompression, we opened the C7 laminae and operate the same procedure as above.

#### 2.4.1. Management and observation following surgery

To prevent infection, antibiotics were given intravenously 24 h before surgery, as well as during the intraoperative and 48-h postoperative periods. The drainage device was removed when the drainage volume was less than 30 ml 24 h after surgery. Patients are discharged from the hospital after they can get out of bed with their neck external fixation and move around on their own and eat normally, usually 3–4 days after surgery. At discharge, the patient was instructed to stay in bed for most of the month, gradually increase the time of sitting and walking, and the neck external fixation must be worn during activities. After 1 month, the patient can no longer wear the neck external fixation and begin functional exercises of the cervical range of motion training. At discharge, the x-ray, CT and MRI results were reviewed. All patients had their x-rays reviewed again about 1 year after surgery.

#### 2.4.2. Statistics and categorization

##### 2.4.2.1. Imagery data

**Figure 2:**
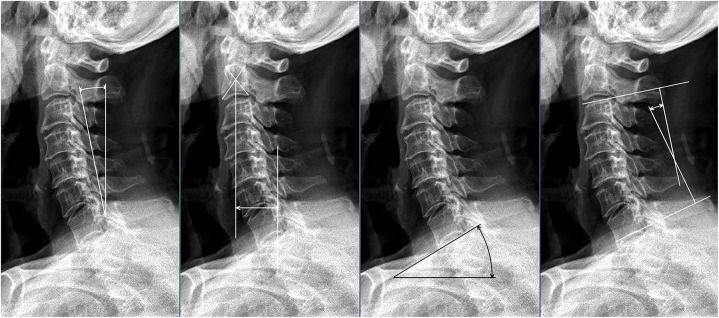
K-line tilt; C2-C7 SVA; T1 slope; CL, respectively.

**Figure 3:**
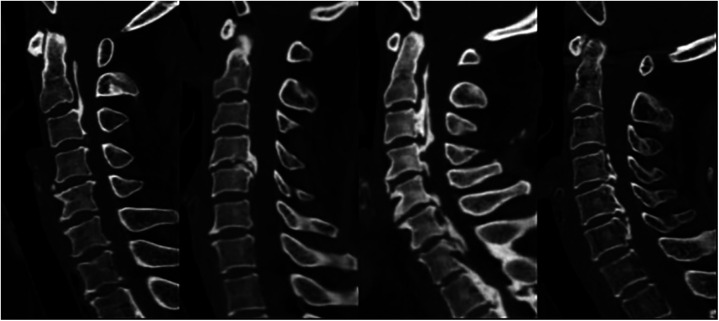
The four types of cervical OPLL: continuous, local, mixed, and segmental.

The patients’ neutral lateral cervical x-rays were analyzed during their hospitalization and 1 year after surgery. The following parameters were measured on x-rays (Figure 2): (1) K-line; (2) K-line tilt (°): the angle formed by the K-line and a line perpendicular to the horizon. (3) C2–C7 SVA (mm): the distance between the vertical line of C2 vertebral body and the posterior upper corner of C7; (4) T1 slope (°): the angle between the upper endplate of the first thoracic vertebra and the horizontal plane; (5) CL (°): cervical curvature, defined as the angle between the superior endplate of C2 and the inferior endplate of C7, <5° was defined as cervical kyphosis, >5° but <10° as straightening of cervical curvature, and >10° as cervical lordosis (18); (6) T1 slope-CL (°): the difference between T1 slope and cervical curvature. Notably, the above parameters were measured at three different time points before being averaged. In addition, the patient's ossification type (continuous, focal, mixed, and segmental) was recorded (Figure 3).

##### 2.4.2.2. Prognostic data

The sagittal x-rays of the cervical spine were reviewed 1 year after the patients’ observation, the presence or absence of cervical kyphosis changes in the patients was recorded, and the patients’ operating segments were recorded. At the 1-year follow-up following discharge, JOA score were collected and expressed as a score to assess neck function. The lower the score, the worse the prognosis. According to the postoperative JOA score of the patients included in the study, the patients were divided into two groups by the median postoperative JOA score: those with a low JOA score with a better prognosis and those with a high JOA score with a worse prognosis.

### 2.5. Statistical evaluation

SPSS 25.0 software was used for statistical analysis (IBM Corp., Armonk, New York, USA). All quantitative data were carried out with homogeneity test of variance, and variables meeting the test were presented as mean ± standard deviation. Independent-samples *T* tests were used for group comparisons. The chi-square test was used for the comparison of counts variables. Correlation tests between different parameters were performed using Pearson correlation test. The relationship between cervical sagittal parameters and postoperative JOA score was analyzed by multiple linear regression analysis. *p* < 0.05 indicated that the difference was statistically significant.

## 3. Results

### 3.1. Fundamental patient information

Table 1 summarizes the basic information of the 50 patients who participated in the study. The average age (years) was 57.46 ± 7.19, with 34 men and 16 women. The type of OPLL could be grouped into: localized 7, segmental 18, continuous 12, and mixed 13. The surgical segments could be grouped into: 3 cases of C2–C6, 39 cases of C3–C6, and 8 cases of C3–C7. The postoperative JOA score of the patients was 12.36 ± 2.02 with a median of 12.5. The K-line tilt (°) was 12.31 ± 5.61, C2–C7 SVA (mm) was 21.10 ± 7.5, T1 slope (°) was 23.03 ± 4.23, CL (°) was 19.31 ± 5.08, and T1 slope-CL (°) was 3.98 ± 6.27.

**Table 1:** Fundamental patient information.

	Mean ± standard deviation
Age	57.46 ± 7.19
Sex	
Male	34
Female	16
Type	
Local	7
Segmental	18
Continuous	12
Mixed	13
Surgical segments	
C2–C6	3
C3–C6	39
C3–C7	8
Postoperative JOA score	12.36 ± 2.02
K-line tilt (°)	12.31 ± 5.61
C2–C7 SVA (mm)	21.10 ± 7.60
T1 slope (°)	23.03 ± 4.23
CL (°)	19.31 ± 5.08
T1 slope-CL (°)	3.98 ± 6.27

### 3.2. Comparison of preoperative and postoperative cervical radiographic parameters, as well as postoperative prognostic parameters

Patients were divided into two groups based on the median postoperative JOA score (12.5): those with a high postoperative JOA score with a better prognosis (*n* = 25; JOA score < 12.5) and those with a low postoperative JOA score with a worse prognosis (*n* = 25; JOA score > 12.5) (Table 2). The cervical radiographic parameters measured by preoperative x-rays were compared between the two groups, as well as postoperative cervical kyphosis changes. In the preoperative period, both groups were K-line (+) and had no cervical kyphosis. There were no significant differences in age, sex ratio, type, or surgical segments between the two groups. In terms of radiographic parameters, there were no significant differences between the two groups in terms of CL, T1 slope-CL, and postoperative kyphosis changes. However, the results of the two groups differed significantly in the comparison of three parameters, K-line tilt, C2–C7 SVA, and T1 slope (*p* = 0.000; *p* = 0.001; *p* = 0.000; and *p* = 0.029).

**Table 2:** Comparison of preoperative and postoperative cervical radiographic parameters, as well as postoperative prognostic parameters.

	Postoperative high JOA score group (*n* = 25)	Postoperative low JOA score group (*n* = 25)	*p*
K-line (+:−)	25:0	25:0	
Lordosis: kyphosis	25:0	25:0	
Age	59.32 ± 7.15	55.60 ± 6.86	0.067
Sex	18:7	16:9	0.544
Type			0.590
Local	2	5	
Segmental	10	8	
Continuous	7	5	
Mixed	6	7	
Surgical segments			0.364
2–6	1	2	
3–6	21	18	
3–7	3	5	
K-line tilt (°)	8.91 ± 4.66	13.71 ± 4.29	0.001
C2–C7 SVA (mm)	17.27 ± 6.50	24.9 ± 6.71	0.000
T1 slope (°)	21.74 ± 3.32	24.32 ± 4.69	0.029
CL (°)	19.57 ± 4.82	19.05 ± 5.41	0.726
T1 slope-CL (°)	2.70 ± 5.79	5.26 ± 6.59	0.151
Kyphosis change	0	0	-

*p *< 0.05 means statistically significant.

### 3.3. Correlation analysis of each cervical radiographic parameter and JOA score

The correlation between cervical sagittal parameters and postoperative JOA score is presented in Table 3. Since these variables were found to be normally distributed, the correlations between cervical radiographic parameters and postoperative JOA score were examined in pairs. The corresponding Pearson correlation coefficients and *p* values were presented, revealing a strong linear relationship between each of the three parameters, K-Line tilt, postoperative JOA score, and C2–C7 SVA (*r* = −0.843, *p* = 0.000; *r* = −0.783, *p* = 0.000; *r* = 0.779, *p* = 0.000), while the linear relationship between T1 slope and postoperative JOA score was moderate (*r* = −0.377, *p* = 0.005) (Table 3).

**Table 3:** Pearson correlation analysis of each cervical radiographic parameter.

	Postoperative JOA score	K-line tilt	C2–C7 SVA	CL	T1 slope	T1 slope-CL
Postoperative JOA score						
Pearson coefficients	1	−0.843**	−0.783**	0.091	−0.377**	−0.314*
P		0.000	0.000	0.55	0.005	0.027
N	50	50	50	50	50	50
K-line tilt						
Pearson coefficients		1	0.779**	−0.072	0.24	0.232
P			0.000	0.617	0.094	0.104
N		50	50	50	50	50
C2–C7 SVA						
Pearson coefficients			1	−0.016	0.19	0.157
P				0.91	0.187	0.276
N			50	50	50	50
CL						
Pearson coefficients				1	0.051	−0.721**
P					0.725	0.000
N				50	50	50
T1 slope						
Pearson coefficients					1	0.590**
P						0.000
N					50	50
T1 slope-CL						
Pearson coefficients						1
P						
N						50

* *p* < 0.05.

** *p *< 0.01.

**Figure 4:**
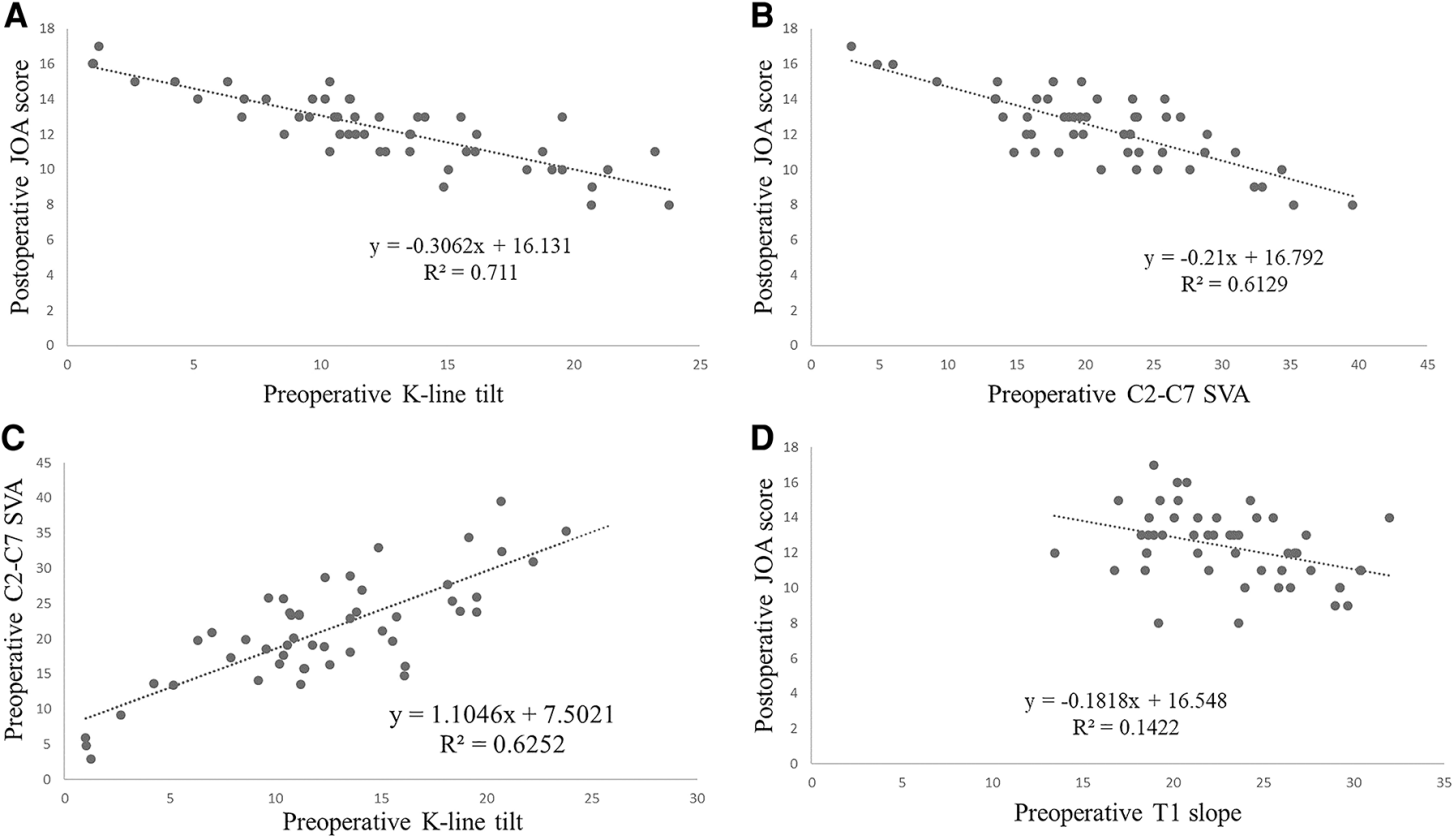
Correlation analysis of each cervical radiographic parameter and JOA score.

Using linear regression analysis, the four groups of variables with linear relationships were plotted and the regression lines labeled. There was a strong linear correlation between the three, K-Line tilt, JOA score, and C2–C7 SVA. *R*^2^ are 0.711, 0.6129, 0.6252 respectively (Figure 4) (note: *R*^2^ is a tabulation that indicates the goodness of fit of the linear regression equation and accepts values ranging from 0 to 1, with values closer to 1 indicating a better fit).

### 3.4. The degree of influence of preoperative K-line tilt, C2–C7 SVA, and T1 slope on postoperative JOA score

As demonstrated in Table 2, the results confirmed that the differences in K-line tilt, T1 slope, and C2–C7 SVA between the two groups were significant, and Table 3 confirmed a strong correlation between postoperative JOA score and the above three. As a result, a multiple linear regression analysis was performed with the above three indicators as independent variables and the postoperative JOA score as the dependent variable, and the results are shown in Table 4 below. Stepwise linear regression analysis yielded the equation *Y* = −0.2X1 −0.086X2 −0.089X3 +18.679 (*Y* = postoperative JOA score; *X*1 = K-line tilt; *X*2 = C2–C7 SVA; *X*3 = T1 slope; *R*^2^ = 0.769). The *R*^2^ values obtained showed that the model was a good fit. The *F* = 55.488 of the regression model, *p* = 0.00 <0.05, proved that the model was statistically significant, and in the significance test of the regression coefficients of respective variables, the corresponding *p*-values of K-line tilt, C2–C7 SVA, and T1 slope were 0.000, 0.005, and 0.012, which are all less than 0.05, proving that all three parameters also possess statistical inference significance. Furthermore, the VIF of these three data sets were 2.602, 1.061, and 2.544, all of which were less than 10, demonstrating no covariance among these three independent variables, and the results of multiple regression analysis were reliable. For the absolute value of the standardized coefficient Beta, K-line tilt > C2–C7 SVA > T1 slope, proving that the influence of these three preoperative imaging parameters on influencing postoperative JOA score was, from the largest to smallest, K-line tilt > C2–C7 SVA > T1 slope, respectively.

**Table 4:** The degree of influence of preoperative K-line tilt, C2–C7 SVA, and T1 slope on postoperative JOA score.

	Unstandardized coefficients B	Unstandardized coefficients std. error	Standardized coefficients beta	*t*	*t* sig. (*p*)	VIF
K-line tilt	−0.2	0.04	−0.550	−4.973	0.000	2.602
C2–C7 SVA	−0.086	0.029	−0.319	−2.916	0.005	2.544
T1 slope	−0.089	0.034	−0.185	−2.614	0.012	1.061
Constant	18.679	0.819		22.801	0.000	

*p *< 0.05 means statistically significant; VIF < 10 means there is no covariance between independent variables.

### 3.5. Case study presentation

**Figure 5:**
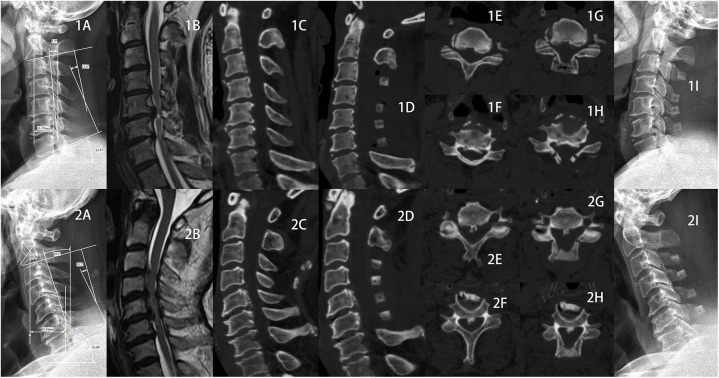
Case Study Presentation 1.

**Figure 6:**
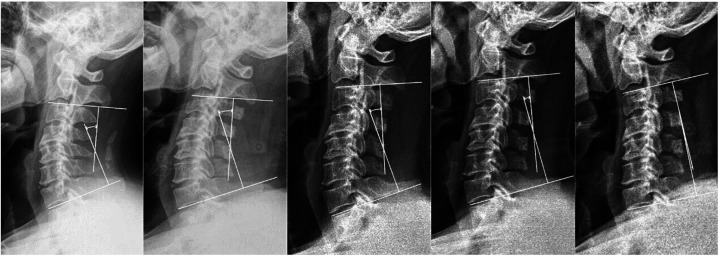
Case Study Presentation 2.

1. Here we present the two typical and comparable patients from the two groups (Figure 5), patient 1 from the high JOA score group and patient 2 from the low JOA score group. In both patients, spinal cord compression was seen on preoperative MRI, both were segmental OPLL, both were K-line (+), both OPLL were located at C5 and C6, and both had enlarged spinal canal volume after lamp. However, they had different postoperative outcomes. Although no abnormalities were seen on x-ray, all preoperative cervical radiographic parameters were generally consistent with the findings indicated by the above results.
2. The Figure 6 depicts x-rays of the cervical spine of a 65-year-old man with cervical OPLL who underwent LAMP and whose CL angles were 26.5°, 21.7°, 17.5°, 10.5°, and −1.8° at 3 days preoperatively, 3, 6, 12, and 24 months postoperatively, respectively. The cervical spine CL angle became negative on the last radiograph, and the cervical spine changed from lordosis to kyphosis.

## 4. Discussion

Studies have shown that K-line (−) or high canal occupying ratio OPLL patients usually have a poor prognosis after LAMP (1, 7, 9–21). The K-line (+) OPLL is the best indication for LAMP, while the K-line (−) OPLL is not. Therefore, only K-line (+) patients were included in this study in order to exclude the biasing effect of lower JOA score of K-line (−) patients on the study results.

This study demonstrated that there was a linear correspondence between K-line tilt and postoperative JOA score. Prior to this, only four studies have been conducted on K-line tilt, Kim (11) found that the patient had severe neck pain when the K-line tilt >25°. Lan (12) found severe neck pain in patients with cervical disc herniation when the K-line tilt >23.75° underwent ACDF. Rao (13) found that the greater the preoperative K-line tilt, the greater the probability of developing postoperative cervical kyphosis change and postoperative neck pain. Sakai (14) found that preoperative K-line tilt >20° was a risk factor for cervical kyphosis change after cervical LAMP. In our study, according to the regression equation established by the postoperative JOA score and the preoperative K-line tilt, the larger the K-line tilt, the lower the postoperative JOA score. This means that when the preoperative K-line tilt is high, OPLL patients have a poor prognosis and severe neck discomfort after 1 year of LAMP treatment.

The following two factors may explain the higher postoperative JOA score in patients with a higher preoperative K-line tilt. For one side, the LAMP procedure is performed by stripping the muscles attached to the cervical spine laminae, thereby exposing and opening the laminae. While the procedure clearly expands the volume of the spinal canal, it disrupts the muscle attachments and cuts the supraspinous and interspinous ligaments, changing the structure of the posterior part of the vertebral body, which are the stabilizing factors that maintain the balance of the cervical spine. The consequence of disrupting these structures is that the muscles of the posterior cervical spine must take on more responsibility and expend more energy to maintain the balance of the head after surgery. For the other side, without considering surgical factors, the higher the K-line tilt, the more the cervical spine tilts forward, the greater the tension required from the posterior cervical muscles, and the more likely the posterior cervical muscles are to fatigue.

This study confirmed a linear relationship between preoperative C2–C7 SVA and preoperative K-line tilt and postoperative JOA score. This is similar to previous findings. Kim (11) and Sakai (14) have also confirmed a linear relationship between C2–C7 SVA and K-line tilt. Furthermore, Tang (22) found a significant positive linear correlation between C2–C7 SVA and NDI score (Neck disability index, an index to assess cervical pain and disability that can be used to assess surgical prognosis).

This study also demonstrated a moderate predictive effect of preoperative T1 slope on postoperative JOA score. Before this study, Xu (23) disclosed that T1 slope, gender, and age were all correlated with the prognosis of cervical spine surgery. Furthermore, Kontt (24) indicated a strong correlation between T1 slope and C2–C7 SVA and that the cervical sagittal is imbalanced when T1-slope was >25° or <13°. However, Cho (25) found that preoperative T1 slope did not aggravate cervical sagittal imbalance. So, controversy remains about the relationship between T1 slope and cervical sagittal balance. In the present study, the preoperative T1 slope significantly differed between the high and low postoperative JOA score groups. It was moderately correlated by linear correlation analysis, while *R*^2^ was too small after listing the linear regression equation, and the equation was not well fitted. In the results analyzed by the multiple linear regression equation, it was demonstrated that T1 slope, although weaker than K-line tilt and C2–C7 SVA, remains an important influence on postoperative JOA score that cannot be ignored.

In the present study, although there was a linear relationship between preoperative T1 slope and CL and T1 slope-CL, there was no linear relationship between these three parameters and K-line tilt. The above reasons can be speculated as follows: T1 slope reflects the degree of kyphosis at the junction of the cervical and thoracic spine, and T1 slope is affected by thoracic spine alignment (26). There are differences in each individual, and although the K-line tilt also varies by thoracic spine alignment, the degree and mechanism by which these two parameters are affected are different.

Patients with LAMP-treated OPLL tend to have postoperative kyphosis change which means poor postoperative prognosis (14). This study had no kyphosis change patient at 1 year after surgery in both the high and low JOA score groups; however, kyphosis change patients was observed at a longer follow-up. Here is a case of a kyphosis change patient (Figure 6), it is hypothesized that the postoperative imbalance in the anterior-posterior muscles tension balance of the cervical spine led to the kyphosis change. In previous studies, Sakai (14) and Lee (27) discovered that a large preoperative K-line tilt is a risk factor for cervical kyphosis change in LAMP treated cervical OPLL. They concluded that a higher K-line tilt meant that the patient's head center of gravity was farther from the midline, and this exacerbated the rate and degree of kyphosis change. They all had a follow-up of more than 2 years. Therefore, we speculate that the lack of correlation between preoperative K-line tilt and postoperative kyphosis change in this study may be due to the short follow-up period in this study.

In summary, it can be concluded that T1 slope has limitations. It is difficult to clearly identify the thoracic spine on lateral x-rays because of rib or sternal occlusion, making it difficult to examine. It is difficult to clearly identify the thoracic spine on lateral x-rays because of rib or sternal occlusion, making it difficult to examine (11). As a length parameter, C2–C7 SVA is easily affected by image magnification and reduction and is difficult to measure if not read in a hospital x-rays reading system. K-line tilt can almost completely solve both problems. K-line tilt is linearly related to C2–C7 SVA, and K-line tilt plays a much larger role in influencing postoperative JOA score than T1 slope and is also easier to measure than it. K-line tilt, as an angular parameter, can be quantified as a linear relationship instead of C2–C7 SVA.

The shortcomings of this study were the small sample size and the fact that it was a single-center study. More importantly, the study did not take into account long-term outcomes. Nevertheless, K-line tilt could be used as a novel cervical sagittal parameter for surgical approach reference and prognostic prediction.

## 5. Conclusion

As preoperative cervical parameters, the influence of K-line tilt, C2–C7 SVA, and T1 slope on postoperative JOA score decreases in order. There was a linear relationship between preoperative K-line tilt and postoperative JOA score, implying that patients with cervical OPLL with high K-line tilt were not eligible for LAMP. K-line tilt was not predictive of cervical kyphosis change after LAMP in patients with OPLL at short-term follow-up.

## Data Availability

The raw data supporting the conclusions of this article will be made available by the authors, without undue reservation.
